# Computed tomographic assessment of lung weights in trauma patients with early posttraumatic lung dysfunction

**DOI:** 10.1186/cc10060

**Published:** 2011-02-25

**Authors:** Andreas W Reske, Alexander P Reske, Till Heine, Peter M Spieth, Anna Rau, Matthias Seiwerts, Harald Busse, Udo Gottschaldt, Dierk Schreiter, Silvia Born, Marcelo Gama de Abreu, Christoph Josten, Hermann Wrigge, Marcelo BP Amato

**Affiliations:** 1 Department of Anesthesiology and Intensive Care Medicine, University Hospital Leipzig, Liebigstrasse 20, D-04103 Leipzig, Germany; 2 Pulmonary Engineering Group, Department of Anesthesiology and Intensive Care Medicine, University Hospital Carl Gustav Carus, Fetscherstrasse 74, D-01307 Dresden, Germany; 3 Department of Trauma and Reconstructive Surgery, University Hospital Leipzig, Liebigstrasse 20, D-04103 Leipzig, Germany; 4 Department of Diagnostic and Interventional Radiology, University Hospital Leipzig, Liebigstrasse 20, D-04103 Leipzig, Germany; 5 Department of Surgery, Surgical Intensive Care Unit, University Hospital Carl Gustav Carus, Fetscherstrasse 74, D-01307 Dresden, Germany; 6 Innovation Center Computer Assisted Surgery (ICCAS), University of Leipzig, Semmelweisstrasse 14, D-04103 Leipzig, Germany; 7 Cardio-Pulmonary Department, Pulmonary Division, Hospital das Clínicas, University of São Paulo Medical School, Av. Dr Arnaldo 455 (Room 2206, 2nd floor), São Paulo 01246-903, Brazil

## Abstract

**Introduction:**

Quantitative computed tomography (qCT)-based assessment of total lung weight (M_lung_) has the potential to differentiate atelectasis from consolidation and could thus provide valuable information for managing trauma patients fulfilling commonly used criteria for acute lung injury (ALI). We hypothesized that qCT would identify atelectasis as a frequent mimic of early posttraumatic ALI.

**Methods:**

In this prospective observational study, M_lung _was calculated by qCT in 78 mechanically ventilated trauma patients fulfilling the ALI criteria at admission. A reference interval for M_lung _was derived from 74 trauma patients with morphologically and functionally normal lungs (reference). Results are given as medians with interquartile ranges.

**Results:**

The ratio of arterial partial pressure of oxygen to the fraction of inspired oxygen was 560 (506 to 616) mmHg in reference patients and 169 (95 to 240) mmHg in ALI patients. The median reference M_lung _value was 885 (771 to 973) g, and the reference interval for M_lung _was 584 to 1164 g, which matched that of previous reports. Despite the significantly greater median M_lung _value (1088 (862 to 1,342) g) in the ALI group, 46 (59%) ALI patients had M_lung _values within the reference interval and thus most likely had atelectasis. In only 17 patients (22%), M_lung _was increased to the range previously reported for ALI patients and compatible with lung consolidation. Statistically significant differences between atelectasis and consolidation patients were found for age, Lung Injury Score, Glasgow Coma Scale score, total lung volume, mass of the nonaerated lung compartment, ventilator-free days and intensive care unit-free days.

**Conclusions:**

Atelectasis is a frequent cause of early posttraumatic lung dysfunction. Differentiation between atelectasis and consolidation from other causes of lung damage by using qCT may help to identify patients who could benefit from management strategies such as damage control surgery and lung-protective mechanical ventilation that focus on the prevention of pulmonary complications.

## Introduction

Trauma patients may be affected by several conditions predisposing them to acute lung injury (ALI) and frequently fulfill all criteria for ALI proposed by the American-European Consensus Conference on Acute Respiratory Distress Syndrome (AECC) [1]. However, concerns have been raised that these ALI criteria (acute onset, presence of a typical risk factor, arterial partial pressure of oxygen to fraction of inspired oxygen ratio (PaO_2_/FiO_2_) less than 300 mmHg, absence of heart failure and bilateral infiltrates visualized on chest X-rays) capture a heterogeneous group of patients and may be nonspecific, particularly in trauma patients [2-4]. The appropriateness of ventilatory management of trauma patients based solely on these criteria has also been questioned [4,5].

Computed tomography (CT) has a higher sensitivity than radiographs for detecting lung parenchymal changes [6,7]. Nevertheless, the visual confirmation of bilateral pulmonary infiltrates by CT instead of chest X-rays is not supported by the current ALI definition and carries the risk of detecting pulmonary opacifications with limited clinical relevance [1,6]. Despite this limitation, quantitative CT (qCT) analysis enables the unique noninvasive assessment of total lung weight (M_lung_) and can be used to distinguish different causes of early posttraumatic pulmonary opacification and thus different populations of ALI patients [2,8-14].

If a patient has pulmonary opacifications on qCT but has a normal M_lung_, atelectasis due to hypoventilation, the use of anesthetics and high inspiratory oxygen concentrations would be the most likely explanation for impaired oxygenation [15]. If a significantly increased M_lung _suggests consolidation from a more significant lung injury (for example, hemorrhage, contusion or edema from capillary leakage) [10-13], a focus on the prevention of secondary lung injury, such as by performing damage control surgery and implementing lung-protective mechanical ventilation, would appear appropriate [3,4,16-19]. Atelectasis mimicking ALI instead may warrant more aggressive ventilatory management and early definitive surgical management [4,5,20-24].

In this study, we aimed to use qCT to (1) establish a reference interval for M_lung _of mechanically ventilated trauma patients with morphologically and functionally normal lungs and (2) study M_lung _in trauma patients who fulfilled the ALI criteria. We hypothesized that qCT would identify atelectasis as a frequent mimic of early posttraumatic ALI. In the future, this information could aid in managing patients with early posttraumatic lung dysfunction.

## Materials and methods

Data for this prospective observational study were collected during routine clinical management at the University Hospital Leipzig. The study was approved by the ethics committee of the University of Leipzig (approval numbers 202/2003 and 311/2007). The need for informed consent was waived because no interventions or additional patient manipulations were required.

**Figure 1:**
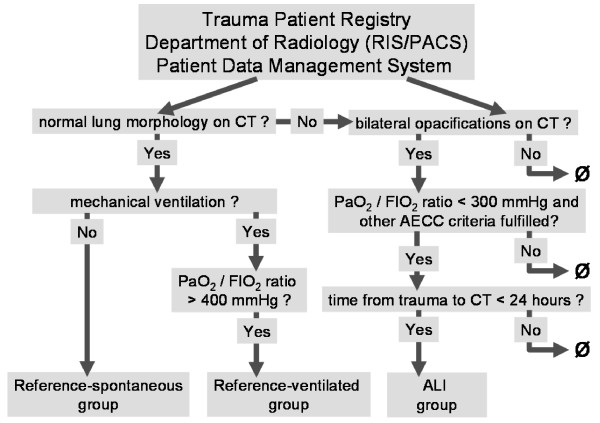
**Flowchart illustrating group assignment**. RIS/PACS, Radiology Information System and Picture Archiving and Communication Systems of the Department of Radiology. CT, computed tomography; PaO_2_/FiO_2_, ratio of arterial partial pressure of oxygen to fraction of inspired oxygen; reference spontaneous group, spontaneously breathing trauma patients with normal lung morphology on CT; reference ventilated group, mechanically ventilated trauma patients with normal lung morphology; ALI group, mechanically ventilated trauma patients fulfilling the criteria for acute lung injury (ALI) as defined by the American-European consensus conference (AECC) on acute respiratory distress syndrome [1]. Ø, exclusion criteria.

Our study consisted of two parts (Figure 1). First, we analyzed the M_lung _of trauma patients with normal lungs to establish a reference interval (reference group). Second, M_lung _values were assessed in patients with early posttraumatic ALI. A small subset of qCT data used in the present study were analyzed in a previous noninterventional study [25].

### Reference group

Trauma patients with morphologically and functionally normal lungs who underwent emergency CT were divided into spontaneously breathing (reference spontaneous) and mechanically ventilated (reference ventilated) patients (Figure 1 and Table 1). Patients with pneumothorax, pleural fluid or opacifications other than small, localized dorsal atelectasis were not included. The decision whether a lung was normal was based on the consensus of one radiologist and two intensivists. If data were available, the PaO_2_/FiO_2 _ratio had to be greater than 400 mmHg.

**Table 1:** Demographic data^a^

Patient demographics	ALI	Reference ventilated	Reference spontaneous
Number of patients	78	43	31
Median age^ns^	42 (23 to 51)	27 (21 to 45)	32 (22 to 44)
Sex (male/female)^b^	61/17	37/6	19/12
Median height^b^, cm	176 (173 to 180)	175 (170 to 183)	174 (168 to 183)
Median weight^b^, kg	80 (74 to 90)	75 (70 to 82)	73 (59 to 85)
Median Body Mass Index^b^, kg m^-2^	26 (23 to 28)	24 (23 to 26)	23 (21 to 24)
Median PaO_2_/FiO_2_, mmHg	169 (95 to 240)	560 (506 to 616)^d^	n.a.
Median Lung Injury Score	2.3 (2.0 to 3.0)	n.a.	n.a.
Median Injury Severity Score^c^	36 (29 to 48)	20 (12 to 26)^d^	12 (6 to 16)^d,e^
Median AIS-T	4 (4 to 4)	n.a.	n.a.
Median Thoracic Trauma Severity Score^b^	11 (9 to 14)	n.a.	n.a.
Median Glasgow Coma Scale score^c^	11 (4 to 15)	11 (7 to 15)	15 (15 to 15)^d,e^
Median volume of intravenous fluids ^c^, ml	2,000 (1,125 to 3,000)	1,000 (500 to 1,500)^d^	1,000 (500 to 1,000)^d^
Median time to CT^ns^, min	122 (90 to 207)	105 (79 to 129)	100 (81 to 136)
Median ventilator-free days^b^	17 (4 to 23)	27 (19 to 27)	n.a.
Median ICU-free days^b^	7 (0 to 17)	22 (10 to 26)	n.a.

^a^All values are given as medians with interquartile ranges. ALI, patients with acute lung injury at admission; reference ventilated, mechanically ventilated patients with normal lungs; reference spontaneous, spontaneously breathing patients with normal lungs; Body Mass Index, weight in kilograms divided by the square of the height in meters; PaO_2_/FiO_2_, ratio of arterial partial pressure of oxygen to fraction of inspired oxygen; AIS-T, Abbreviated Injury Scale of the Thorax; time to CT, interval between trauma and computed tomography (CT); ventilator-free days, number of days without mechanical ventilation within a period of 28 days; ICU, intensive care unit; ICU-free days, number of days without ICU treatment within a period of 28 days; n.a., not applicable; ^ns^, not significant. Positive end-expiratory pressure (PEEP) was 10 cmH_2_O in all mechanically ventilated patients except for five; in three patients, PEEP >10 cmH_2_O was already applied before admission and two patients were spontaneously breathing during CT. ^b^No statistical test performed. ^c^*P *< 0.001 for the Kruskal-Wallis test over all groups. ^d^*P *< 0.001 versus ALI. ^e^*P *< 0.05 versus reference ventilated group.

### ALI group

Trauma patients were eligible for the ALI group if they had undergone CT within 24 hours posttrauma, fulfilled the clinical criteria for ALI (that is, acute onset, typical trigger, absence of heart failure and PaO_2_/FiO_2 _ratio below 300 mmHg) at admission and CT showed bilateral pulmonary opacifications (Figure 1) [1].

Physiological and demographic data were obtained from the patient data management system into which these data had been prospectively and automatically entered. The ventilator-free days and the intensive care unit (ICU)-free days were calculated as the number of days without mechanical ventilation or ICU treatment, respectively, within a period of 28 days [26]. The Lung Injury Score (LIS), the Injury Severity Score (ISS), the Abbreviated Injury Scale of the Thorax (AIS-T) and the Thoracic Trauma Severity Score (TTSS) were calculated at the time of admission [27-29]. The Glasgow Coma Scale (GCS) score at the trauma scene and the amount of intravenous fluids administered prior to CT were calculated on the basis of the ambulance report form.

Pressure-controlled mechanical ventilation (reference ventilated and ALI) during primary resuscitation and CT was standardized and included the following ventilator settings (Oxylog 3000; Dräger, Lübeck, Germany): target tidal volume of 6 ml/kg estimated body weight (estimated weight in kilograms equals height in centimeters minus 100), respiratory rate of 20 breaths min^-1 ^and positive end-expiratory pressure of 10 cmH_2_O [21,30].

### CT scanning

Each CT scan was requested by the treating physicians as routine diagnostic procedure in emergency trauma patients [21,31]. Depending on availability, two multislice CT scanners were used, either a Somatom Volume Zoom (120-kV tube voltage, 165-mA tube current, 4 × 2.5-mm collimation; Siemens, Erlangen, Germany) or a Philips MX8000 IDT 16 (120-kV tube voltage, 170-mA tube current, 16 × 1.5-mm collimation; Philips Medical Systems, Hamburg, Germany). As part of routine clinical imaging, contiguous images were reconstructed with either 10-mm slice thickness and the enhancing filter "B60f" on the Siemens scanner or 5-mm thickness and the standard filter "B" on the Philips scanner. Intravenous with contrast material (120 ml of iopamidol 300; Schering, Berlin, Germany) was used as part of the clinical protocol in all patients. Because of the observational study design, the degree of inspiration during CT could not be controlled: Reference spontaneous patients were asked to hold their breath after inspiration (without checking for compliance) during CT. Reference ventilated and ALI patients were scanned during uninterrupted mechanical ventilation, which is current clinical practice in our institution. Calibration of the CT scanners was performed using air and the manufacturer's standard phantom.

### Quantitative CT analysis

The lung parenchyma was segmented manually in CT images covering the entire lungs (Osiris software; University Hospital Geneva, Geneva, Switzerland) [25]. Window levels and widths appropriate for the lung parenchyma (-500/1,500 HU) or the mediastinum (50/250 HU) were used. Major hilar vessels and bronchi, pneumothoraces, pleural fluids and gross motion artefacts were manually excluded. Only in aerated lung regions did we use a threshold (-350 HU)-based segmentation technique in an attempt to guide and standardize the manual exclusion of partial volume effects close to the thoracic wall, mediastinum, heart or diaphragm. To do so, window level and width were set to (-350/0 HU), and the segmentation line was drawn at the black-white interface [32-34]. Opacified lung regions were segmented manually using anatomical landmarks.

The total lung volume (V_lung_), the total lung mass (M_lung_) and the masses of differently aerated lung compartments were calculated voxel-by-voxel using customized software as previously described [9,10,12,25,35]. M_lung _and V_lung _values were calculated on the basis of all lung voxels within the -1,000 to +100 HU range. The following HU ranges were used to separate differently aerated lung compartments: nonaerated, -100 to +100 HU; poorly aerated, -101 to -500 HU; normally aerated, -501 to -900 HU; and hyperaerated, -901 to -1,000 HU. The masses of differently aerated lung compartments were calculated as percentages of M_lung_. Although it was calculated, we omitted between-group comparison of the hyperaerated compartment because two different CT scanners and image reconstruction protocols were used, and such comparison was not required for the present study [30].

The validity of our analytical method was reviewed in 27 patients by placing a water-filled plastic bottle next to the thorax. We then selected an arbitrary region of interest (ROI) within this bottle in the CT image and compared the weight resulting from our voxel-by-voxel analysis method with that obtained by simply multiplying the volume of interest (ROI area × slice thickness) by the volumetric mass density of water (approximately 997.77 kg/m^3 ^at 22°C).

### Statistical analysis

Data are given as medians with interquartile ranges unless specified otherwise. According to Clinical and Laboratory Standards Institute guideline C28-A3 [36], the 95% reference interval of M_lung _was calculated using the robust method because the number of reference subjects was smaller than 120 [36,37]. Results were compared between subgroups using the Mann-Whitney *U *test or the Kruskal-Wallis test. Confidence intervals (95% CI) for normal M_lung _reported in previous studies were calculated [38]. Analysis of variance (ANOVA) was used to compare the M_lung _values from these previous studies with our reference patients (Shapiro-Wilk test indicated normal distribution). Linear regression analysis was used to calculate coefficients and 95% CIs for the correlation of body height and weight with M_lung_. The effect of adjusting for sex, age and group regarding the relationship between M_lung _and body height was tested by entering these variables into the regression model. It was defined *a priori *that only variables explaining ≥5% of the variance in M_lung _values would be kept in the final model. Bland-Altman plots were used to compare the ROI weights used for validation of our voxel-by-voxel analytical method [39]. All tests were two-sided. Statistical significance was assumed if *P *< 0.05. Statistical analyses were performed using SPSS 12.0 software (SPSS, Inc., Chicago, IL, USA) and MedCalc software (MedCalc Software, Mariakerke, Belgium).

## Results

### Reference patients

We analyzed 74 trauma patients with morphologically and functionally normal lungs. Reference ventilated patients were more frequently male, more severely injured and received more intravenous fluids than reference spontaneous patients. One reference ventilated patient (2%) died as a result of severe head injury. Demographic data are given in Table 1.

Results from qCT are given in Table 2. Supporting their classification as normal, all reference patients had negligible amounts of nonaerated lung (Table 2). The median M_lung _of all reference patients was 885 (771 to 973) g, and the mean M_lung _of all reference patients was 871 (95% CI, 838 to 905) g. The 95% reference interval for M_lung _was 584 to 1,164 g. No significant differences (*P *= 0.55; ANOVA) were found between mean M_lung _values of reference ventilated, reference spontaneous or mean normal M_lung _reported by Gattinoni *et al*. [10] (850 (95% CI, 785 to 915) g), Puybasset *et al*. [11] (943 (95% CI, 857 to 1,029) g) and Whimster *et al*. [40] (850 (95% CI, 818 to 881) g).

**Table 2:** Lung volumes and weights quantified by CT^a^

Parameter	ALI	Reference ventilated	Reference spontaneous
Median V_lung_^b^, ml	3,208 (2,574 to 4,289)	4,228 (3,701 to 4,621)	3,195 (2,670 to 4,918)
Median V_lung _in women^b^, ml	2,865 (2,413 to 3,293)	3,498 (2,957 to 3,948)	2779 (2,526 to 3,878)
Median V_lung _in men^b^, ml	3,304 (2,562 to 4,513)	4,426 (3,801 to 4,760)	3363 (2,979 to 6,121)
Median M_lung_^c^, g	1,088 (862 to 1,342)	893 (785 to 968)^d^	884 (724 to 986)^d,e^
Median M_lung _in women, g	814 (748 to 1,250)	738 (664 to 765)	720 (620 to 824)
Median M_lung _in men, g	1,119 (913 to 1,358)	902 (847 to 981)	928 (864 to 993)
Median M_hyper_^b^, %	0 (0 to 3)	2 (0 to 4)	0 (0 to 4)
Median M_normal_^b^, %	55 (39 to 68)	88 (85 to 91)	85 (79 to 88)
Median M_poor_^b^, %	17 (14 to 23)	6 (6 to 10)	9 (7 to 17)
Median M_non_^b^, %	20 (11 to 34)	1 (1 to 2)	1 (1 to 2)

^a^All values are given as medians with interquartile ranges. ALI, patients with acute lung injury already at admission; reference ventilated, mechanically ventilated patients with normal lungs; reference spontaneous, spontaneously breathing patients with normal lungs; V_lung_, total lung volume; M_lung_, total lung mass; M_hyper_, mass of hyperaerated lung compartment; M_normal_, mass of normally aerated lung compartment; M_poor_, mass of poorly aerated lung compartment; M_non_, mass of nonaerated lung compartment. The weights of differently aerated lung compartments were calculated as percentages of M_lung_. V_lung _and M_lung _values were calculated for each sex separately as well as for all patients in a group to assess sex-specific differences. ^b^Because the degree of inspiration was not controlled during computed tomography, between-group comparison of V_lung _and differently aerated lung compartments was omitted. ^c^*P *< 0.001 for the Kruskal-Wallis test over all groups; ^d^*P *< 0.001 versus ALI; ^e^*P *= 0.74 versus reference ventilated.

For reference patients, M_lung _correlated moderately with body height (*R*^2 ^= 0.35, *P *< 0.0001), but not reliably with actual body weight (*R*^2 ^= 0.14). The equation for the regression of M_lung _(in grams) on body height (in centimeters) for all reference patients had the following parameters: coefficient (height) = 9.3 (95% CI, 6.4 to 12.3) and *y*-intercept = -768 (95% CI, -1291 to -246). Adjustment for sex by including a dummy-coded sex variable (male = 0) significantly improved the model for regression of M_lung _on body height (Δ*R*^2 ^= 0.05, *P *= 0.02 for the *R*^2 ^change). The parameters of the sex-adjusted regression equation were coefficient (height) = 7.2 (95% CI, 3.8 to 10.6), coefficient (sex) = -88.6 (95% CI, -160.7 to -16.5) and *y*-intercept = -365 (95% CI, -973 to 244). Adjusting for age or group (reference spontaneous versus reference ventilated) did not improve the model (*P *= 0.65 and *P *= 0.14, respectively).

### ALI patients

Seventy-eight patients fulfilled the AECC criteria for ALI at admission. All patients were severely injured, and only one patient (ISS = 12) had an ISS below 16 points. Demographic data are given in Table 1, and the results of qCT are given in Table 2.

Fifteen ALI patients (19%) died as a result of nonpulmonary complications, nine patients died of severe head injury, five died of uncontrollable hemorrhage and one died of late sepsis and multiorgan failure. Patients who died did not have greater M_lung _than survivors (*P *= 0.75). Patients with severe head injury (GCS score <8, *n *= 30) [41] had significantly greater M_lung _(1,274 (962 to 1,634) g) than patients with GCS score ≥8 (*n *= 48, 981 (802 to 1,161) g; *P *< 0.001).

Although the median M_lung _(1,088 (862 to 1,342) g) of our ALI patients was significantly greater than that of our reference patients (*P *< 0.0001), it was lower than the mean values reported for other ALI patients, for example by Patroniti et al. (1,513 (95% CI 1,426 to 1,600) g) and by Gattinoni et al. (1,500 (95% CI 1,380 to 1,620) g) [10,12,42].

No reliable correlation was found between M_lung _and scores for trauma severity (ISS, AIS-T, TTSS, LIS and GCS), the volume of intravenous fluids, the PaO_2_/FiO_2 _ratio or the time between trauma and CT (all *R*^2 ^≤ 0.16).

**Figure 2:**
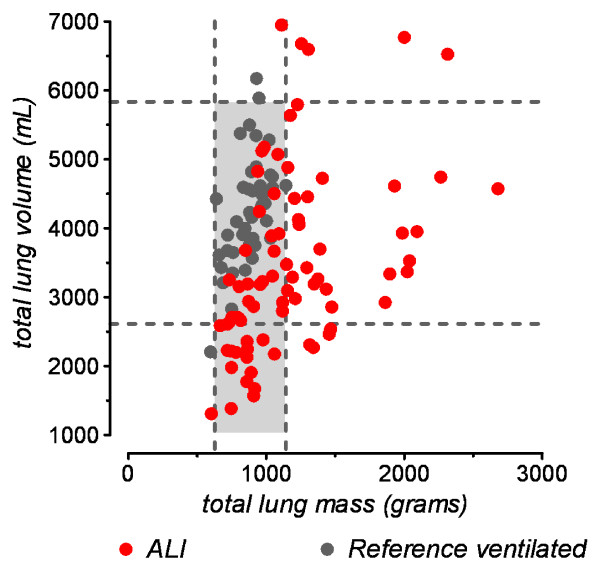
**Comparison of lung weights**. Lung weights of 78 patients with acute lung injury (ALI) upon admission (red circles) in comparison to the values of 43 mechanically ventilated trauma patients with morphologically and functionally normal lungs (reference ventilated, gray circles). Dashed lines mark the 95% reference intervals for total lung mass and total lung volume, respectively, calculated from reference ventilated patients. Because reference ventilated patients were ventilated with the same positive end-expiratory pressure (10 cmH_2_O) and also underwent computed tomography during uninterrupted mechanical ventilation, only these reference ventilated patients were used for the graphical comparison with ALI patients in this graph. ALI patients whose data points fall within the gray box did not have an increased lung weight.

Forty-six (59%) ALI patients had M_lung _below the upper limit of the reference interval (that is, 1,164 g) and were thus allocated to an atelectasis subgroup (Figure 2, Table 3). We also defined a consolidation subgroup using the lower limit of the 95% CI of the mean M_lung _(i.e. 1380 g) reported for ALI patients by Gattinoni *et al*. [10]. Statistically significant differences between atelectasis and consolidation patients were found for the parameters age, LIS, GCS, V_lung_, mass of the nonaerated lung compartment and, interestingly, ventilator-free days and ICU-free days (Table 3).

**Table 3:** Patient subgroups defined by different ranges of lung weights^a^

Patient subgroups	Atelectasis (≤ reference range)	Above reference range	Consolidation
Definition	M_lung _≤1,164 g	M_lung _>1,164 g	M_lung _>1,380 g
Number of patients^b^	46 (59%)	32 (41%)	17 (22%)
Median age^c^, yr	45 (32 to 53)	28 (17 to 46)	21 (17 to 48)
Median PaO_2_/FiO_2_^ns^, mmHg	184 (128 to 252)	136 (78 to 238)	132 (68 to 230)
Median Lung Injury Score^e^	2.3 (1.6 to 2.6)	2.7 (2.3 to 3.3)	3.0 (2.3 to 3.3)
Median Injury Severity Score^ns^	34 (29 to 45)	41 (28 to 50)	36 (25 to 50)
Median AIS-T score^b^	4 (4 to 4)	4 (4 to 4)	4 (4 to 4)
Median Thoracic Trauma Severity Score^ns^	11 (8 to 14)	12 (9 to 15)	12 (11 to 15)
Median Glasgow Coma Scale score^e^	14 (10 to 15)	6 (3 to 12)	7 (3 to 15)
Median volume of intravenous fluids ^ns^, ml	2,000 (1,000 to 3,000)	2,000 (1,500 to 2,875)	2,500 (1,500 to 3,000)
Median time to CT^ns^, min	135 (90 to 220)	112 (90 to 177)	131 (103 to 227)
Median ventilator-free days^d^	19 (10 to 25)	15 (0 to 19)	15 (0 to 19)
Median ICU-free days^c^	14 (2 to 22)	1 (0 to 13)	5 (0 to 14)
Median V_lung_^e^, ml	2,832 (2,226 to 3,669)	3,812 (3,134 to 4,696)	3,696 (3,019 to 4,668)
Median M_lung_^b^, g	899 (787 to 1,048)	1,398 (1,265 to 1,972)	1,930 (1,461 to 2,065)
Median M_non_^e^, %	16 (10 to 25)	34 (18 to 52)	40 (33 to 57)

^a^All values are given as medians with interquartile ranges. Atelectasis, patients with lung weights (M_lung_) within the reference interval (that is, 584 to 1,164 g) for normal M_lung_; above reference, patients with M_lung _values exceeding the upper limit of the reference interval (that is, 1,164 g); consolidation, patients with M_lung _values exceeding the lower limit of the 95% confidence interval of the mean M_lung _values reported for patients with acute lung injury (that is, 1,380 g [10]); PaO_2_/FiO_2_, ratio of arterial partial pressure of oxygen to fraction of inspired oxygen; AIS-T, Abbreviated Injury Scale of the Thorax; time to CT, interval between trauma and computed tomography (CT); ventilator-free days, number of days without mechanical ventilation within a period of 28 days; ICU, intensive care unit; ICU-free days, number of days without ICU treatment within a period of 28 days; V_lung_, total lung volume; M_lung_, total lung mass; M_non_, percentage mass of nonaerated lung compartment (percentage of M_lung _value); ^ns^, not significant. ^b^No statistical test performed. ^c^*P *< 0.05, ^d^*P *< 0.001 and ^e^*P *< 0.01, respectively, for the Kruskal-Wallis test over all groups.

### Validation of the mass estimation technique

The mean (± standard deviation) weight of the test-ROI obtained by geometrical calculation was 13.0 ± 5.4 g. The values from our voxel-by-voxel method were slightly smaller. The mean difference (bias) between both methods was -2.4% and the limits of agreement were -4.6% and 0.2% of the mean weight of the test-ROI.

## Discussion

We found that atelectasis was the most likely cause of lung dysfunction in more than half of patients who fulfilled the clinical criteria for ALI and showed lung opacifications on admission CT early after trauma.

Comparison of M_lung _values derived from qCT with a reference interval for normal M_lung _could help to assess the etiology of ALI and improve the definition of different populations of ALI patients [2,8,10-14,42]. A group of mechanically ventilated, volume-loaded trauma patients with morphologically and functionally normal lungs offered us the opportunity to confirm the normal range of M_lung _obtained in previous analyses of diagnostic CT in healthy, spontaneously breathing volunteers [10,11]. The M_lung _values measured in our reference groups are in good agreement with the M_lung _values from these previous qCT analyses and M_lung _of normal lungs at autopsy [10,11,40]. Thus, our results suggest that moderate positive intrathoracic pressure potentially affecting pulmonary blood and/or lymph flow and moderate intravenous volume loading have limited effect on M_lung_.

Calculation of M_lung _and parameters such as the excess lung tissue or weight by performing qCT can help to distinguish atelectasis from consolidation due to more significant lung damage [10-13,43]. It could be argued that atelectasis may also be distinguished visually from contusion or edema on the basis of typical topographical distributions. Analysis of qCT, however, can still assess M_lung _in the presence of pleural fluid or when atelectasis is obscuring edema or pulmonary contusions [16,22]. When lung aeration is impaired without a concomitant increase in M_lung_, atelectasis is the most likely explanation [11,13]. Accordingly, atelectasis was the most plausible cause of lung dysfunction in 59% of our ALI patients (Table 3). Interestingly, atelectasis patients also had significantly lower V_lung _values than consolidation patients (Table 3). Although V_lung _was not controlled in our study, the latter observation is compatible with the concept of atelectasis: V_lung _is reduced by collapse, while consolidation of the lung does not necessarily decrease V_lung _[44]. The identification of trauma patients in whom atelectasis mimics ALI could be helpful in decision making and individualization of care (that is, early definitive stabilization rather than damage control surgery). Atelectasis may persist into the posttraumatic period, promote bacterial growth and nosocomial pneumonia and affect patient outcome [3,23,45-50]. Therefore, more aggressive ventilatory management, early definitive surgical treatment and timely weaning from mechanical ventilation could shorten the ICU treatment and reduce the incidence of infections in patients with atelectasis [4,20-24,49].

Thirty-two ALI patients (41%) had increased M_lung_. In only 17 patients (22%) was M_lung _increased to the range previously reported for ALI patients, suggesting consolidation from more significant lung injury due to contusion, hemorrhage, aspiration or edema resulting from pulmonary and/or systemic inflammation with capillary leakage [10-13]. Although fluid overload may also play a role [3], we did not find significantly higher infusion volumes in consolidation patients, and all five patients who received more than four liters of infusions had M_lung _values within the reference interval (Table 3). The association of severe head injury with increased M_lung _further underlines the fact that multiple factors, such as neurogenic pulmonary edema, may be involved in the development of posttraumatic lung dysfunction [41]. Even if the precise etiology of posttraumatic lung dysfunction remains unclear in some patients, information on preexisting lung damage could help clinicians to judge the individual patient's tolerance for further aggressive shock resuscitation and definitive surgical repair [20,24]. It could also guide clinicians in choosing treatment concepts such as lung-protective mechanical ventilation or damage control surgery, which are focused on the prevention of "second hits" to lungs which have already been primed by shock and pulmonary or systemic injuries. Among such "second hits" are surgical trauma, ongoing intraoperative blood loss and transfusion, fat embolism following intramedullary nailing or injurious mechanical ventilation [3,17-20,51].

Parameters such as ISS or PaO_2_/FiO_2_, which have previously been used for the prediction and further characterization of posttraumatic ALI, failed to distinguish atelectasis from consolidation patients [3,52,53]. In contrast, age as well as LIS, GCS and qCT results differed statistically significantly between these groups. Interestingly, atelectasis patients spent fewer days on mechanical ventilation and in the ICU than consolidation patients (Table 3). However, given the fact that all patients fulfilling the ALI criteria early after trauma have been managed according to the damage control concept in our institution, the latter differences should be considered hypothesis-generating rather than hypothesis-confirming. The variable reliability of clinical parameters and scores for characterizing posttraumatic ALI supports the potential clinical usefulness of qCT, which is the only available *in vivo *method to directly and reliably quantify M_lung _and the amount of nonaerated lung tissue, which both characterize the severity of lung injury [10-12,52].

Some aspects of our methodology warrant discussion. (1) We studied ALI patients within 24 hours after trauma (Table 1) because it was our aim to study the etiology of early posttraumatic respiratory failure, which may differ significantly from respiratory problems developing later [3,4,49,54]. (2) All whole-body CT scans performed in our emergency trauma patients routinely involved the clinically indicated application of contrast material [21,31]. A possible effect of contrast material on the normal M_lung _was the reason why we included a reference group and did not refer only to existing data [10,11,40,55]. The normal M_lung _found in our reference patients matched that in previous reports, which supports the lack of an effect of contrast material on the qCT assessment of M_lung _in patients with normal lungs [55]. Patients with atelectasis should also remain unaffected by a possible contrast material-associated increase in M_lung_. In contrast, the leakage of contrast material into the pulmonary interstitium may artefactually increase M_lung _calculated on the basis of qCT in patients with an injured alveolar-capillary barrier [55]. However, although desirable from a scientific perspective, contrast material administration appears unavoidable in emergency trauma patients, and a possible artefactual increase in M_lung _must be taken into account. (3) Because varying segmentations result in inconsistent M_lung _values, we used a threshold-based (-350 HU) segmentation technique in addition to manual segmentation to improve the highly subjective manual exclusion of partial volume effects at the boundaries of aerated lung regions. So far, no CT study in ALI patients has included such attempts, and thus this threshold was adopted from other thoracic qCT applications. (4) Because the manual interaction necessary for qCT analysis is time-consuming, it might still be considered unrealistic to introduce qCT-based information into clinical practice. The extrapolation method, which we described recently, offers significant time savings and could aid the clinical implementation of qCT [14,25].

### Limitations of our study

Because chest X-rays were not obtained in addition to CT scans during routine clinical imaging, we could not confirm the presence of infiltrates conventionally on the basis of chest X-rays. Moreover, our results may not be directly transferrable to patients subjected to higher intrathoracic pressures or massive intravenous volume loading. While M_lung _is only minimally affected, parameters characterizing lung aeration and volume depend on the degree of inspiration as well as on differences between CT scanners and image reconstruction protocols. Because CT scanning was performed during ongoing mechanical ventilation, the end-expiratory amount of nonaerated lung might have been underestimated. Different CT scanners and image reconstruction interact with the quantification of hyperaeration. Therefore, we omitted the between-group comparison of the differently aerated lung compartments, which was not the focus of the present study (Table 2) [30].

## Conclusions

qCT can detect different etiologies of posttraumatic lung dysfunction. Atelectasis was the most likely cause of early posttraumatic lung dysfunction in more than half of our patients. Whether individualized care based on qCT actually offers an option to prevent secondary lung injury, reduce posttraumatic pulmonary complications and improve outcome remains to be studied.

## Key messages

• Diagnosis, management and further study of ALI in trauma patients may be hampered by uncertainties about the fulfillment of the criteria for ALI proposed by the AECC.

• Differentiation between atelectasis and consolidation of the lung by qCT may help to identify patients with different etiologies of posttraumatic lung dysfunction.

• In our study, atelectasis was the most likely cause of early posttraumatic lung dysfunction in more than half of patients, and only 20% of patients had M_lung _values in the range previously reported for ALI.

• Trauma patients with atelectasis may require shorter periods of mechanical ventilation and treatment in the ICU.

• In the future, information from qCT could aid in managing patients with early posttraumatic lung dysfunction.

## Abbreviations

AECC: American-European Consensus Conference on Acute Respiratory Distress Syndrome; AIS-T: Abbreviated Injury Scale of the Thorax; ALI: acute lung injury; ANOVA: analysis of variance; ARDS: acute respiratory distress syndrome; 95% CI: 95% confidence interval; CT: computed tomography; FiO_2_: fraction of inspired oxygen; GCS: Glasgow Coma Scale; HU: Hounsfield units; ICU: intensive care unit; IQR: interquartile range; ISS: Injury Severity Score; LIS: Lung Injury Score; M_lung_: lung weight; PaO_2_: arterial partial pressure of oxygen; PEEP: positive end-expiratory pressure; qCT: quantitative analysis of computed tomography; TTSS: Thoracic Trauma Severity Score; V_lung_: lung volume.

## Competing interests

The authors declare that they have no competing interests.

## Authors' contributions

AWR and APR contributed equally to this work. AWR, APR, DS, MS, CJ and MBPA planned the study. AWR, APR, DS, MS, HB, and UG were responsible for the data acquisition. AWR, APR, TH, AR, MS, HB, SB and UG performed the quantitative CT analysis. AWR, PMS, HW, MGA and MBPA undertook the statistical analysis. All authors contributed to the preparation of the manuscript. The principal investigators, AWR and APR, had full access to the data analyzed in the study and take full responsibility for the integrity of all of the data and the accuracy of the data analysis.
